# Identification of CD66c as a potential target in gastroesophageal junction cancer for antibody–drug conjugate development

**DOI:** 10.1007/s10120-025-01584-z

**Published:** 2025-02-07

**Authors:** Peng Zhang, Changjuan Tao, Hanfei Xie, Liu Yang, Ye Lu, Yun Xi, Shili Yao, Li Yuan, Peng Guo, Xiangdong Cheng

**Affiliations:** 1 Department of Gastric Surgery, Zhejiang Cancer Hospital, Hangzhou Institute of Medicine (HIM), Chinese Academy of Sciences, Hangzhou, China; 2 Zhejiang Provincial Research Center for Upper Gastrointestinal Tract Cancer, Zhejiang Cancer Hospital, Hangzhou, China; 3 Zhejiang Key Lab of Prevention, Diagnosis and Therapy of Upper Gastrointestinal Cancer, Zhejiang Cancer Hospital, Hangzhou, China; 4 Department of Radiation Oncology, Zhejiang Cancer Hospital, Hangzhou, 310022 Zhejiang China; 5 Department of Gynecologic Oncology, Zhejiang Cancer Hospital, Postgraduate Training Base Alliance of Wenzhou Medical University, Hangzhou, 310022 Zhejiang China; 6 Present Address: Department of Medical Oncology, Zhejiang Provincial People’s Hospital, Hangzhou, 310022 Zhejiang China; 7 Clinical and Translational Research Center, Hangzhou Institute of Medicine (HIM), Chinese Academy of Sciences, Hangzhou, 310022 China; 8 Department of Pathology, Zhejiang Cancer Hospital, Hangzhou, 310022 Zhejiang China; 9 School of Materials Science and Engineering, Tianjin University, Tianjin, 300072 China

**Keywords:** CD66c, Gastroesophageal junction cancer, Antibody–drug conjugate, Targeted therapy

## Abstract

**Background:**

Gastroesophageal junction (GEJ) cancer exhibits unique biological characteristics and currently lacks specific targeted therapies. Given the clinical efficacy of antibody–drug conjugates (ADCs) in solid tumor treatment, we aimed to identify a novel ADC target and suitable payload for GEJ-targeted therapy.

**Methods:**

In this study, we conducted bioinformatic analyses of multi-omics data, including transcriptomics, proteomics, and phosphoproteomics, to identify CD66c as a promising ADC target for GEJ cancer. We then engineered a CD66c-directed antibody–drug conjugate (CD66c-DXd) incorporating a GGFG linker. The preclinical efficacy of CD66c-DXd was determined in multi GEJ xenograft models.

**Results:**

Proteomic analyses of 103 cases of GEJ cancer revealed that CD66c expression was significantly higher in tumoral tissues compared to normal tissues. Proteomic and phosphoproteomic analyses identified deruxtecan (DXd) as a potentially potent payload for ADCs targeting GEJ cancer. Furthermore, high CD66c expression in GEJ was associated with a significantly lower proportion of plasma cells. The drug-to-antibody ratio (DAR) of CD66c-DXd was determined to be 3.6. CD66c-DXd effectively and selectively ablated multiple human GEJ cell lines (OE-19, OE33 and SK-GT-4) without affecting non-malignant cells (GES-1) in vitro. Eventually, CD66c-DXd mediated potent and durable tumor regression in vivo with excellent safety profiles.

**Conclusions:**

This preclinical study provides a strong rationale for the further development of CD66c-DXd as promising therapeutic candidates to treat advanced GEJ cancer. Additionally, the study demonstrates the robustness of the multi-omics data in identifying novel potential ADC targets and payloads.

## Introduction

Gastroesophageal junction (GEJ) cancer is a lethal malignancy defined as adenocarcinomas arising within a 5 cm range of the esophagogastric junction [1]. The incidence of GEJ has been rapidly increasing during the past few decades [2, 3]. Due to anatomical proximity and pathological similarities of GEJ cancer, the ongoing clinical trial often grouped GEJ cancer under gastric cancer. However, epidemiological studies have indicated distinct risk factors between the two malignancies [4, 5]. Furthermore, large-scale retrospective studies have revealed that GEJ cancer exhibits different clinical manifestations and pathological characteristics compared to gastric cancer, with a higher incidence of recurrence and significantly poorer prognosis [6, 7]. Therefore, it is imperative to consider GEJ cancer as a separate entity from both esophageal cancer and gastric cancer. Currently, the main treatment of GEJ cancer is surgery-based multidisciplinary therapy. Unfortunately, most GEJ cancer patients are diagnosed at advanced stages with locally advanced tumors or distant metastases, rendering them ineligible for surgery. Targeted therapies are only suitable for a limited population (10–22%) of patients with advanced HER2-positive malignancy [8]. The objective response rate of pembrolizumab (a PD-L1 antibody) monotherapy was only 11.6% (30/259) in advanced gastric cancer and GEJ cancer patients [9]. Therefore, there remains a pressing unmet clinical need to discover new molecular targets and develop novel targeted therapeutics for GEJ cancer treatment.

Antibody–drug conjugates (ADCs) have emerged as a promising therapeutic approach for both hematological tumors and solid malignancies. Known as “magic bullets,” ADCs combine the specificity of monoclonal antibodies with the cytotoxic potency of small molecule drugs. This unique mechanism of ADC has shown potential for improving the efficacy and safety of cancer therapies in the clinic. Promising results have been reported from several clinical trials using ADCs for the treatment of advanced-stage gastric cancer. Trastuzumab deruxtecan, an ADC targeting HER2, has received FDA approval for treating advanced HER2-positive gastric cancer [10]. Other ADC targets under investigation in clinical trials and preclinical studies for gastric cancer include HER3, guanylyl cyclase C (GCC), Trop-2, and CLDN18.2 [11–14]. However, to date, there has been no comprehensive multi-omics study specifically screening cell membrane proteins as ADC targets for GEJ.

Our previous study provided an integrative proteomic characterization of 103 patients with GEJ cancer [15], identifying three distinct proteomic subtypes of GEJ cancer. Leveraging these in-house proteomic data, in this study, we performed an unbiased screening of specific membrane targets for GEJ cancer using both proteomic and RNA sequencing data. We then compared the cell surface abundance of conventional cancer targets with the newly identified targets. Moreover, we selected a suitable payload for ADC synthesis based on phosphorylation modifications identified in phosphoproteomic data. This study establishes a strong foundation for the development of a promising ADC to address the critical needs in GEJ cancer patient care.

## Materials and methods

### Cell lines, cell culture, and siRNA silencing

Human GEJ cell lines, including OE19 (Cat. CBP60495, OE19 cell line was derived from a 72-year-old male patient with gastric cardia adenocarcinoma in 1993), OE33 (Cat. CBP60496, OE33 cell line was derived form a 73 years old female patient with adenocarcinoma of lower esophagus), and SK-GT-4 (Cat. CBP60462, SK-GT-4 cell line was derived from the primary tumor of an 89-year-old Caucasian male with an adenocarcinoma of the distal esophagus in 1989), and normal human gastric mucosal epithelial cells GES-1 (Cat. CBP60512) were obtained from Cobioer Biosciences Co., Ltd (Nanjing, China). All cell lines were identified by short-tandem-repeat profiling and were negative for bacterial and fungi contamination. OE19 and SK-GT-4 cells were cultured in Roswell Park Memorial Institute (RPMI)-1640 containing 10% fetal bovine serum and 1% penicillin/streptomycin. GES-1 cells were cultured in DMEM containing 10% fetal bovine serum and 1% penicillin/streptomycin. Human peripheral blood mononuclear cells (PBMCs) were isolated and maintained in RPMI 1640 supplemented with 10% FBS. All cells were maintained at 37 °C in a humidified incubator with 5% (vol/vol) CO_2_. CD66c silencing was by siRNA knockdown. CD66c-targeted siRNA was transfected in OE19 and OE33 cells, using jetPRIME^®^ according to the manufacturer’s protocol. The CD66c siRNA sequences are as follows: 5′-CCUGCACAGUACUCUUGGUUUTT-3′.

### Bioinformatic analysis

The Gene Ontology (GO) enrichment analysis and Reactome pathway analysis enrichment of DEGs were conducted between CD66c-high and CD66c-low groups. The CIBERSORT and xCELL algorithms were employed to deconvolute bulk RNA-seq profiles and quantify the infiltration of immune cells, fibroblasts, and other cell types in different CD66c expression groups. The CD66c expression in TCGA cohort was assessed in Gene expression Profiling Interactive Analysis 2 (GEPIA2) (http://gepia2.cancer-pku.cn) [16]. The GEJ cancer external validation cohort (MSK cohort; 902 samples [17]) was analyzed by cBio Cancer Genomics Portal (http://cbioportal.org) [18]. The GEJ cancer was classified as Chromosomal instability (CIN) if they were microsatellite stable and FACETS-corrected fraction of genome altered (FGA) ≥ 5% as previously described [19, 20]. The single cell external validation dataset (GSE134520) was analyzed with IMMUcan scDB (https://immucanscdb.vital-it.ch) [21, 22].

### Hematoxylin–eosin (HE) staining, immunohistochemistry (IHC) staining, multiplex immunofluorescence (mIF) staining, and methylene Blue staining

A series of formalin-fixed, paraffin-embedded GEJ cancer tissues (*n* = 95), adjacent normal tissue (*n* = 49), and lymph-node metastasis samples (*n* = 48) were collected in Zhejiang Cancer Hospital as previously described to produce TMA [15]. IHC staining of serial TMAs for CD66c expression was conducted as described previously [23]. In briefly, slides were blocked with 5% normal goat serum buffer at 37 °C for 30 min after a 10-min treatment with 3% H_2_O_2_/methyl alcohol solution. Slides were subsequently incubated overnight at 4 °C with primary antibody (CD66c, 1:500; Cat#ab275022, Abcam). After thorough washing, slides were incubated with biotin labeled goat anti-rabbit IgG and HRP-conjugated streptavidin for 1 h at 37 °C. Immunoreaction was visualized by incubation with diaminobenzidine (DAB) (Cat#ZLI-9065, ZSGB-BIO). Then, slides were counterstained with hematoxylin (Cat#ZLI-9609, ZSGB-BIO) dehydrated and subsequently blocked. The stained slides were independently evaluated by two experienced pathologists. IHC staining of CD8a and CD138 in tumor samples from mice was performed as mentioned above. Brown-stained cells were regarded as positive. The expression of CD66c, CD8a, and CD138 was assessed using the H-score system and the formula for the H-score was as described previously [15]. Methylene Blue staining was performed to detect *Helicobacter pylori* (*HP*) according to the manufacturer’s instructions (Beso, China).

### Evaluating cell surface CD66c expression and its internalization

Cell membrane expression of CD66c were measured by CytoFLEX LX Flow Cytometer (Beckman Coulter) as previously reported [24]. In brief, a total of 10^6^ cells were collected and then rinsed twice via suspension–spin cycles with ice-cold PBS. Blocking was accomplishing with 1% bovine serum albumin (BSA) for 30 min in an ice bath. After BSA blockage, cells were incubated with phycoerythrin (PE) conjugated CD66a, CD66c, and CD66e antibodies (ab275673, ab42796, ab275676; Abcam) for 1 h at room temperature. Stained cells were rinsed with 1%BSA-PBS three times, resuspended in PBS for flow cytometry.

The antibody internalization experiment was done as previously described [23]. Briefly, 2 × 10^6^ cells were first incubated with unconjugated anti-CD66c antibodies (ab78029; Abcam) for 30 min in an ice bath. The stained cells were rinsed with 1%BSA-PBS and resuspended in PBS. The unconjugated primary CD66c antibodies bound on cell membranes were allowed to undergo internalization at different time points (0 min, 30 min, 60 min, 120 min, and 240 min). Subsequently, secondary PE-conjugated rat anti-mouse IgG (BioLegend, San Diego, CA) was added to the stained cell for 30 min and then rinsed by PBS, and fixed with 4% paraformaldehyde for flow cytometry. The internalization efficiency was calculated by an established formula (1-mean cell fluorescence intensity (*t* = incubation time)/mean cell fluorescent intensity (*t* = 0 min)) × 100% [25, 26]. The internalization curve was generated base on the internalization efficiency at different time points.

### Immunofluorescent (IF) staining

Cells were cultured on a confocal dish overnight. Then, PE-conjugated CD66c antibody was incubated with cells at 37 °C for 45 min, washed with pre-cold PBS, fixed with 4% paraformaldehyde, blocked by 1% BSA, and analyzed by confocal microscopy (Nikon A1 HD25; Nikon, Japan). For internalization experiments, cancer cells were incubated with PE-conjugated CD66c antibody as described and washed with pre-cold PBS, and subsequently were visualized using a Nikon fluorescence microscope after different time points (0 min, 30 min, 60 min, 120 min, and 240 min). Cell nuclei were counterstained with Hoechst 33258 in PBS for 10 min at room temperature.

### Preparation and characterization of ADC targeting CD66c

For the preparation of the ADC targeting CD66c, tinurilimab (BAY1834942, Cat# HY-P99051, Bayer, Germany), as an anti-CD66c antibody commercially available, was selected [27]. The linker and payload of ADC targeting CD66c was MC-GGFG-DXd (Cat#GC35840, GLPBIO, USA) with by potent anti-tumor activity using DXd (an inhibitor of DNA topoisomerase I), linked via the protease cleavable MC-GGFG linker [28]. The synthesis of CD66c-ADC was prepared as described previously [29]. In brief, the BAY1834942 was reduced with a tenfold molar excess of tris(2-carboxyethyl) phosphine (TCEP, Catalog No. GC10529) at 37 °C for 1 h. After adjusting the pH, incubating and filtering, the reduced BAY1834942 was then linked with tenfold molar equivalents of MC-GGFG-DXd (Catalog No. GC35840) at 15 °C overnight with gentle shaking. The free MC-GGFG-DXd and potential precipitates were removed by Ultra-4 Centrifugal Filter (30 K MWCO). The drug-to-antibody ratio (DAR) of ADC targeting CD66c was measured using matrix-assisted laser desorption ionization time-of-flight mass spectrometry (MALDI-TOF) [30].

### Evaluation of cell cytotoxicity

Human GEJ cells were seeded in a 96-well plate at a density of 6000 cells per well overnight. The cell culture medium was replaced with a medium containing either chemotherapy drugs (paclitaxel; maximum concentration: 117.11 μmol/L) or CD66c-DXd (maximum concentration: 0.67 μmol/L) at serially diluted concentrations. Cell cytotoxicity was assessed after 96 h using a CCK-8 kit (KeyGEN Biotech, China). The absorbance at 450 nm was measured using an ELISA browser (Bio-Tek EL 800, USA). The experiments were repeated thrice.

OE19, OE33, and SK-GT-4 cells were seeded into 96-well plates and treated accordingly. After 48 h of treatment, the cells were harvested and rinsed with PBS (pH 7.4). Subsequently, the cells were fixed with 2.5% phosphate-buffered glutaraldehyde for 1 h at 4 °C before embedding. The cells were then post-fixed with 1% osmium tetroxide for 30 min, dehydrated using a gradient of increasing ethanol and acetone, and embedded in epoxy resin following three PBS washes.

The GEJ cancer cells were supplemented with fresh medium and co-cultured with PBMCs in the presence of IL-2 (200 U/mL) in a ratio of 1:5 (cancer cell: PBMCs). The co-culture model was treated with CD66c-DXd (10 nM) or CD66c mab (10 nM) for 96 h. After incubation and treatment, the cells were collected for flow cytometry analysis. Primary antibodies against PE-CD138 (ab128936, Abcam) and APC-CD45 (ab28106, Abcam).

### In vivo evaluation of anti-tumor efficacy of CD66c-DXd in xenograft mouse models

The subcutaneous mouse tumor model was constructed by injection of 10^7^ OE19 tumor cells as previously described [23]. The tumor-bearing nude mice were randomly divided into three groups (*n* ≥ 5 per group) and administered treatment with PBS, paclitaxel, or CD66c-DXd at a consistent dosage of 5 mg/kg/week via tail vein injection. Tumor growth was assessed twice weekly through two-dimensional measurements using a vernier caliper, while the bodyweight and size of the tumor were monitored thrice weekly. Tumor volume was calculated according to the formula: tumor volume (mm^3^) = tumor width^2^ × tumor length × 0.5. The tumor-bearing mice were euthanized at the end of the study, or when tumors reached 1500 mm^3^.

To generate GEJ cancer patient-derived xenograft (PDX), fresh GEJ cancer fragments were transplanted subcutaneously (s.c.) into the right flank of anaesthetized NCG mice (male, 4–5 weeks old).

### Statistical analysis

Quantitative data are presented as means ± SD. Two-tailed Student’s *t* test was applied when comparing two means. Comparison of multiple groups was performed using one-way or two-way ANOVA. Afterward, one-way ANOVA or two-way ANOVA with Bonferroni post hoc correction was applied and adjusted *p* value was defined as the uncorrected *p* value multiplied by the number of comparisons. Statistical analysis was performed using Prism 9 (Graph Pad Software Inc.). *p* values <0.05 were considered statistically significant.

## Results

### Identification of CD66c as a potential target for advanced GEJ cancer

**Fig. 1:**
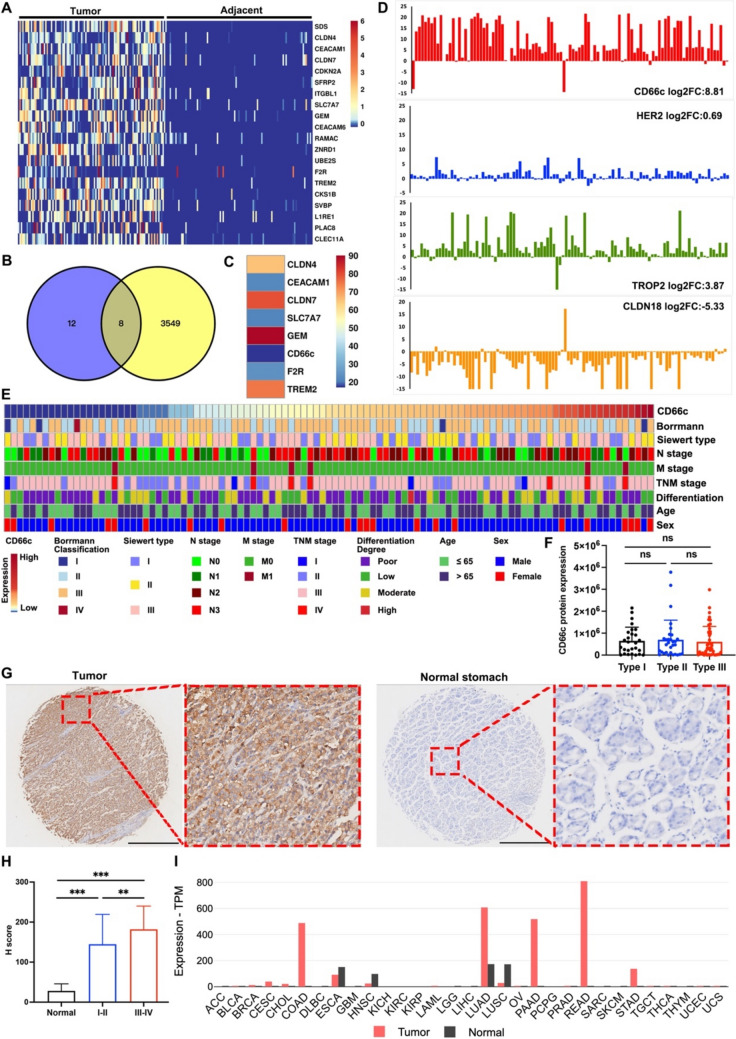
CD66c was overexpressed in GEJ cancer. **A** Heatmap of top 20 DEPs between AEG tumor and paired NAT samples; **B** Venn diagram of top 20 DEPs and membrane proteins from Cancer Surfaceome Atlas; **C** heatmap of the normal tissue expression index of eight candidate targets; **D** bar chart of log_2_ fold-change of established ADC targets (HER2, TROP2, and CLDN18) and CD66c in the tumor and precancerous tissues in each GEJ patient; **E** heatmap of clinicopathological characteristics among different CD66c expression level; **F** box plots compare CD66c protein expression quantities in different Siewert types; **G** representative images of IHC staining of CD66c in human normal gastric tissue, primary GEJ, and metastatic lymph node; **H** H-score of CD66c among adjacent normal tissue and different TNM stages; **I** the CD66c mRNA expression between paracancerous tissues and cancers from TCGA database. *** *p* < 0.001; ** *p* < 0.01; * *p* < 0.05

To meet the safety and efficacy criteria for ADC targets, ADC targets should be abundantly presented on the cell surface of tumors with undetectable levels in normal tissues [29, 31]. Our previous study reported the proteomics and phosphoproteomics profiling of 103 GEJ tumors with paired normal adjacent tissues (NATs) and RNA sequencing in 83 tumor-NAT pairs. Figure 1A presents the top 20 differenced expression protein (DEPs) based on the proteomics data of 103 AEG tumors. Next, we cross-referenced the Cancer Surfaceome Atlas [32] and found that 8 of the top 20 DEPs were localized to cell membrane (Fig. 1B). We also compared the expression of these 8 proteins in human normal tissues using the Human Protein Atlas (HPA). To quantify and compare the normal tissue expression of different targets, we defined the normal tissue expression index, which is the sum of the expression scores across 41 tissue expression scores from HPA, with a high expression score of 3, a middle expression score of 2, a low expression score of 1, and a non-expression score of 0. Figure 1C displays the normal tissue expression index of 8 protein and the index of CD66c was the lowest. We also compared CD66c with other established ADC targets (HER2, TROP2, and CLDN18.2) for GEJ. Since CLDN18.2 was the isoform of CLDN18 and specifically expressed in gastric tissue [33], the expression of CLDN18.2 in GEJ was represented by CLDN18. Figure 1D illustrates the histogram of log_2_ fold-change in the tumor and precancerous tissues of each GEJ cancer patient, demonstrating that the tumor expression specificity of CD66c was the highest among tested target candidates. Figure 1E shows the heatmap representation of the clinical characteristics in relation to CD66c expression levels of 103 GEJ cancer patients, indicating that CD66c expression increased significantly with advanced TNM stage. In the 103 GEJ cancer patients, there were 27 Siewert type I patients, 31 Siewert type II patients, and 45 Siewert type III patients. The protein-level of CD66c was similar in different Siewert types (Fig. 1F). To further validate our proteomics findings, we performed an IHC staining of CD66c in tissue microarrays of GEJ cancer cohort (Fig. 1G). The IHC results revealed that immunostaining of CD66c was predominantly membranous and normal gastric tissue samples had no/low expression of CD66c (Fig. 1G). As is shown in Fig. 1H, the IHC H-score of GEJ patients with stage III–IV was significantly higher than that of patients with stage I–II stage and normal gastric tissue. On the other hand, we also assessed whether overexpressed CD66c expression was broadly observed in other cancer types in TCGA cohort, which included 33 cancer types. As is shown in Fig. 1I, CD66c is particularly overexpressed in pan-gastrointestinal malignancies and lung adenocarcinoma (LUAD). In the normal tissue group serving as the control, CD66c is consistently low/negatively expressed. Specially, in stomach adenocarcinoma (STAD) cohort, CD66c expression in cancer group was significantly compared with normal group. Collectively, these data provide support for the conclusion that CD66c represents a potential ADC target candidate for advanced GEJ cancer.

### CD66c is associated with immunosuppressive phenotype of GEJ cancer

**Fig. 2:**
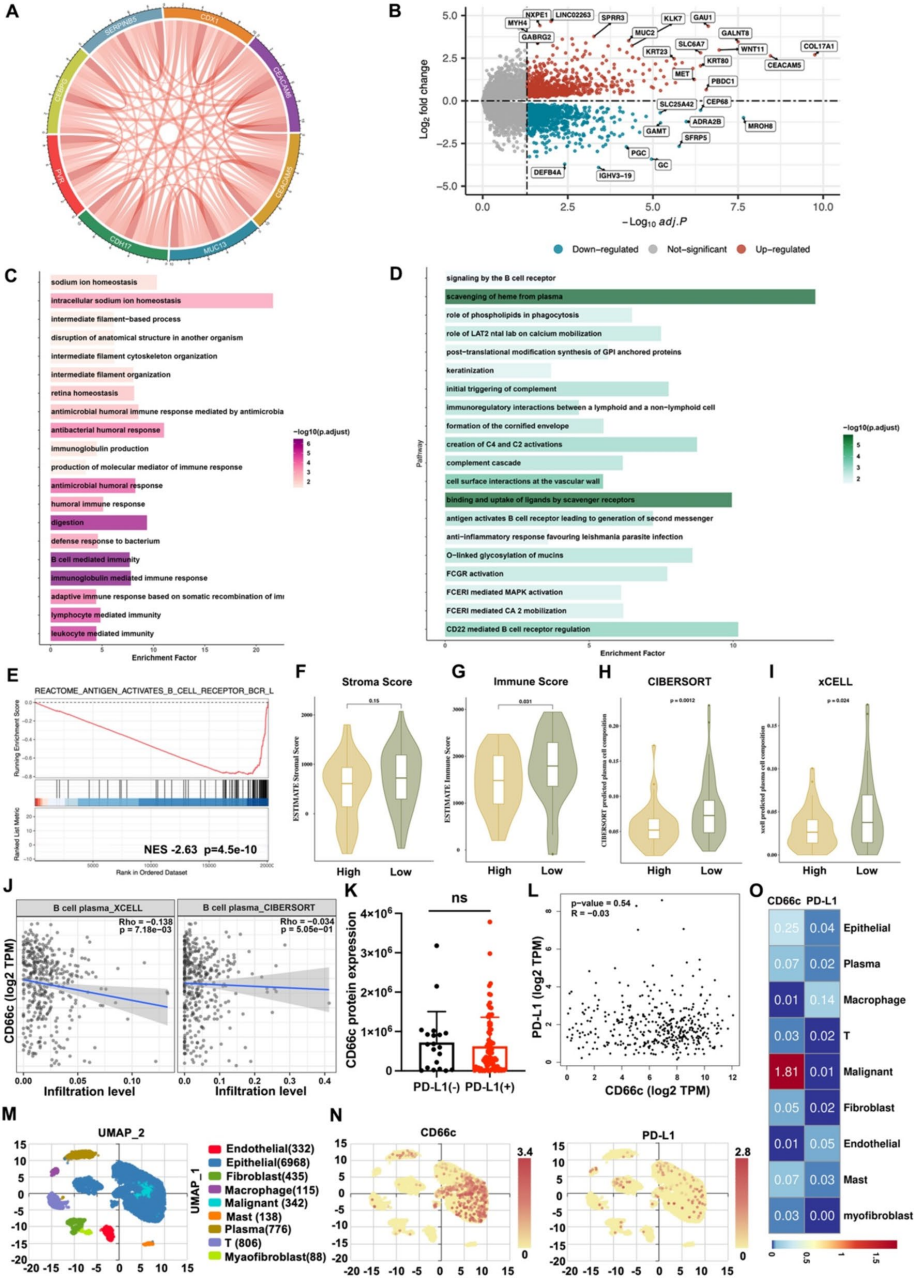
Immune cell landscape and CD66c expression in GEJ patients. **A** Pie chart of the genes most closely related to CD66c; **B** volcano map of high-CD66c expression versus low-CD66c expression group; **C** the top 30 pathways of Gene Ontology (GO) enrichment analysis; **D** the top 30 pathways of Reactome pathway analysis; **E** antigen activates B-cell receptor leading to the generation of second messengers was significantly suppressed in high-CD66c expression group; **F** stroma score estimated by ESTIMATE between high-CD66c expression group and low-CD66c expression group; **G** immune score estimated by ESTIMATE between high-CD66c expression group and low-CD66c expression group; **H** proportion of plasma cells estimated by CIBERSORT between high-CD66c expression group and low-CD66c expression group; **I** proportion of plasma cells estimated by xCELL between high-CD66c expression group and low-CD66c expression group; **J** the correlations between CD66c and plasma cells in TCGA STAD cohort; **K** the CD66c protein expression between PD-L1(−) group and PD-L1(+) group; **L** the correlations between CD66c mRNA expression and PD-L1 mRNA expression in TCGA STAD cohort; **M** uniform manifold approximation and projection (UMAP) of the single cell dataset (GSE134520), colored by cell-type; **N** UMAP plot of CD66c and PD-L1 expression in different cell types; **O** the mean expression level of CD66c and PD-L1 in different cell types

To investigate the biological role of CD66c in GEJ tumors, we conducted a transcriptomic analysis on 83 paired in-house GEJ cancer samples. This analysis identified genes closely associated with CD66c, including CDX1, MUC13, CDH17, PVR, CEBPG, SERPINB5, and CEACAM5, many of which are involved in forming immunosuppressive microenvironment (Fig. 2A). A volcano plot (Fig. 2B) illustrates the differential gene expression between high and low CD66c expression groups, with the cut-off value set at the median expression level of CD66c. Gene Ontology (GO) enrichment analysis (Fig. 2C) and Reactome pathway analysis (Fig. 2D) both indicated significant suppression of B-cell-mediated immunity in the CD66c-high group. Notably, the antigen activation of the B-cell receptor was markedly inhibited in the CD66c-high group (NES = −2.63, *p* < 0.001; Fig. 2E). Next, to explore the roles that the CD66c played in tumor microenvironment has during tumor development, we estimated immune score and stroma score using the ESTIMATE algorithm. The ESTIMATE analysis revealed that the stroma score was similar between high-CD66c expression group and low-CD66c expression group, while the immune score was significantly lower in high-CD66c expression group compared with low-CD66c expression group (stroma score *p* = 0.15, immune score *p* = 0.031; Fig. 2F, G). To corroborate these findings, multiple immune deconvolution algorithms (CIBERSORT and xCELL) were manipulated to estimate the abundances of immune cells infiltrating into GEJ. The CIBERSORT analysis revealed that the proportion of plasma cell (also called effector B cell or antibody-secreting cell) in high-CD66c expression group was significantly lower than low-CD66c expression group (*p* = 0.001, Fig. 2H). This reduction was also confirmed by the xCELL algorithm (*p* = 0.024; Fig. 2I). The association between CD66c and plasma cells was externally validated by TCGA STAD cohort. The xCELL algorithm revealed that CD66c expression was significantly negatively associated with plasma cells (*r* = −0.138, *p* = 0.007; Fig. 2J). The CD66c expression was also negatively associated with plasma cells with the CIBERSORT algorithm and the difference was not significantly (*r* = −0.034, *p* = 0.505; Fig. 2J). Considering the essential role of PD-L1 in tumor immune escape, we also explored the correlation between CD66c and PD-L1. There was no correlation between CD66c and PD-L1 protein expression (*r* = −0.09; *p* = 0.35) in the proteomics of 103 GEJ cancer patients. Besides, the CD66c protein expression level was similar between PD-L1 negative group (PD-L1 negative was defined as the PD-L1 protein level was 0) and PD-L1 positive group (*t* = 0.52, *p* = 0.61; Fig. 2K). There was also no correlation between CD66c and PD-L1 expression in the TCGA STAD cohort as the external validation (*r* = −0.03; *p* = 0.54; Fig. 2L). We also explored the CD66c and PD-L1 expression distribution in single cell dataset (GSE134520) with IMMUcan Database [21, 22] (Fig. 2M). UMAP plot showing expression distribution of CD66c and PD-L1 in different cell types (Fig. 2N). The CD66c gene expression was dominantly distributed in malignant cells and the PD-L1 expression was dominantly distributed in macrophage cells (Fig. 2O). These comprehensive multi-omic analyses support the conclusion that CD66c is associated with an immunosuppressive microenvironment in GEJ cancer. The immunosuppressive microenvironment represented by CD66c was independent of immune escape mediated by PD-L1.

**Fig. 3:**
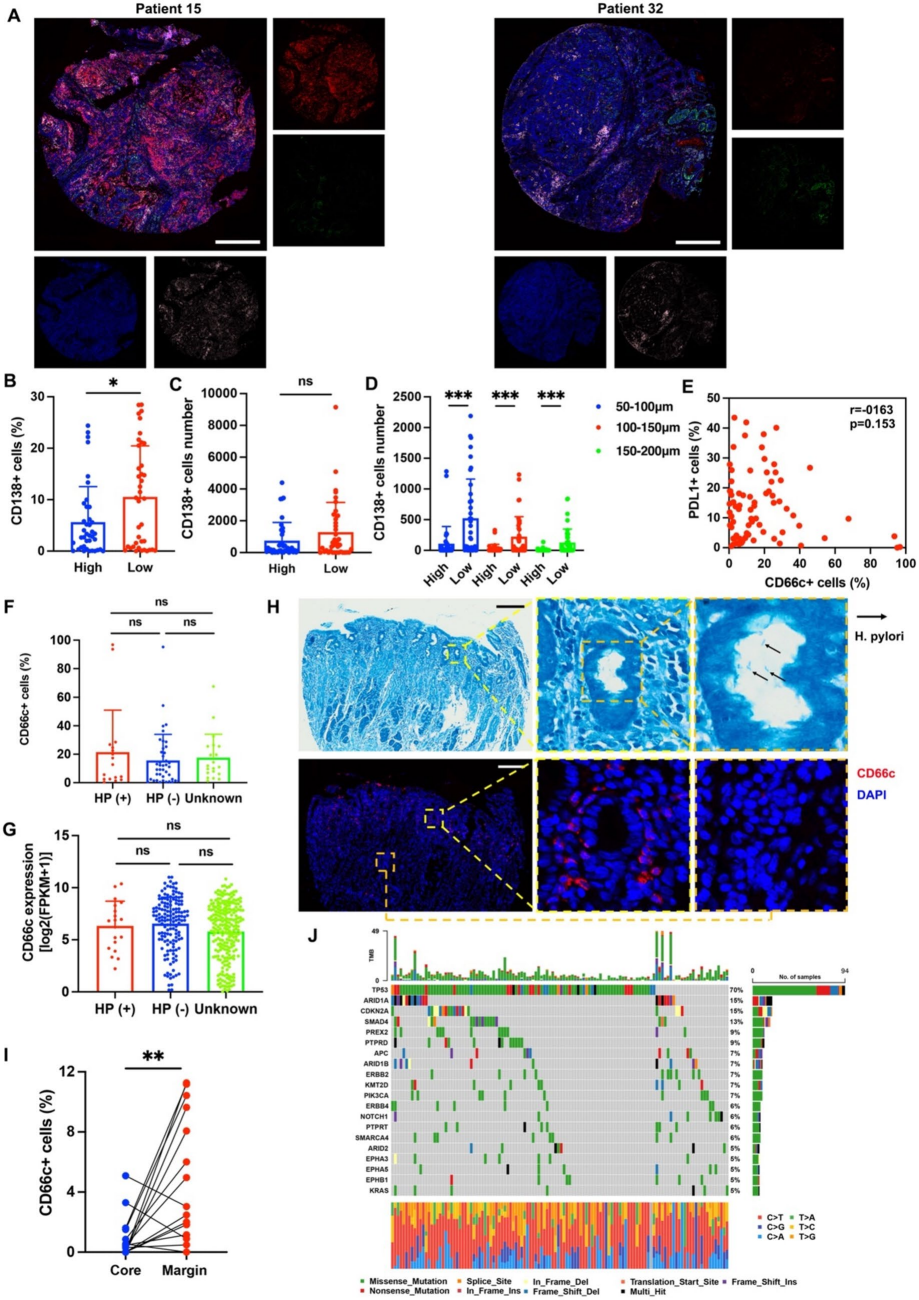
The association among CD66c positivity and plasma cells and *H. pylori* infection in vivo. **A** Representative mIF images of 78 GEJ cancer patients; **B** the proportion of CD138+ cells between CD66c high and CD66c low group; **C** the number of CD138+ cells at distance of 0–50 μm between CD66c high and CD66c low group; **D** the number of CD138+ cells at distance of 50–100 μm; 100–150 μm; 150–200 μm between CD66c high and CD66c low group; **E** the correlations between CD66c+ cells and PD-L1+ cells in 8 GEJ cancer patients; **F** the proportion of CD66c+ cells among HP(+); HP(−) and Unknown groups in 78 GEJ cancer patients; **G** the mRNA expression of CD66c among HP(+); HP(−) and Unknown groups in TCGA STAD cohort; **H** representative staining image of *H. pylori* infection and corresponding mIF image of CD66c; scale bar 200 μm; black arrows indicated *H. pylori*. **I** The proportion of CD66c+ cells between mucosa margin and mucosa core group; **J** the oncoplot generated with maftools depicting the top 20 mutated cancer-related genes of 141 GEJ cancer patients in MSK cohort

The association between CD66c expression and plasma cells was further externally validated by independent clinical samples (78 GEJ patients) with multiplex immunofluorescence (mIF) (Fig. 3A). The mIF results revealed that the proportion of CD138+ (marker of plasma cells) cells in CD66c high group (cut-off value: median) was significantly lower than in CD66c high group (5.63% vs. 10.53%; *p* = 0.013; Fig. 3B). The intensity of CD138+ cells was also calculated at different radius (0–50 μm; 50–100 μm; 100–150 μm; 150–200 μm) from CD66c+ cells. As is shown in Fig. 3C, the number of CD138+ cells at distance of 0–50 μm from CD66c+ cells in CD66c high group was lower than that in in CD66c high group, but the difference was not statistically significantly (mean number 755.2 vs. 1286.0; *p* = 0.133). The number of CD138+ cells at distance of 50–100 μm from CD66c+ cells in CD66c high group was significantly lower than that in in CD66c high group (mean number 108.4 vs. 523.5; *p* < 0.001; Fig. 3D). The similar results were obtained at distance of 100–150 μm and 150–200 μm (100–150 μm: *p* < 0.001; 150–200 μm; *p* = 0.001; Fig. 3D). On the other hand, the number of PD-L1+ cells is not related to the number of CD66c+ cells (*r* = −0.163, *p* = 0.153; Fig. 3E).

Considering the important role of Helicobacter pylori (*H. pylori*) infection in pathogenesis of gastric cancer and GEJ cancer, we explored the potential association between CD66c expression and *H. pylori* infection. In the 78 GEJ cancer patients with mIF, there were 17 patients with *H. pylori* infection. The status of *H. pylori* infection of 41 patients was diagnosed as negative. The status of *H. pylori* infection in the rest 20 patients was unknown. As is shown in Fig. 3F, the proportion of CD66c+ cells between *H. pylori* infection group (HP+) and negative *H. pylori* infection group (HP-) was similar. The TCGA STAD cohort was setted as the external validation. In TCGA STAD cohort, there were 19 patients with *H. pylori* positive; 150 patients with *H. pylori* negative and 238 patients with unknown infection status. The CD66c expression level in *H. pylori* infection group (HP+) and negative *H. pylori* infection group (HP-) was also similar (Fig. 3G). The majority of *H. pylori* reside in semi-permeable mucous gel layer of the stomach blanketing the apical surface of gastric mucosa epithelium [34]. Thus, we also assessed the CD66c positive cell rate in different distances from gastric mucosa (mucosa margin vs. mucosa core) based on the paracancer gastric tissues of 15 GEJ cancer patients. As is shown in Fig. 3H and I, the proportion of CD66c+ cells was significantly higher in mucosa margin compared with that in mucosa core (*p* = 0.004).

We also assessed the associations with frequent genetic mutations in GEJ cancer and chromosomal instability (CIN). The largest sample dataset of esophagogastric cancer to date (MSK cohort; 902 samples) [17] was applied. In the 902 patients, we reviewed the gastroesophageal locations of cancer and retrieved 141 samples that were regarded as GEJ in the MSK cohort [17]. In the 141 patients, 115 patients were subtyped as CIN. The oncoplot of 141 GEJ cancer patients is shown in Fig. 3J. The top 10 mutated genes were TP53, ARID1A, CDKN2A, SMAD4, PREX2, PTPRD, APC, ARID1B, ERBB2, and KMT2D. The association analysis revealed that GEJ cancer patients with CIN had a significantly lower prevalence of ARID1A mutation.

### CD66c is a potential ADC target for GEJ cancer

**Fig. 4:**
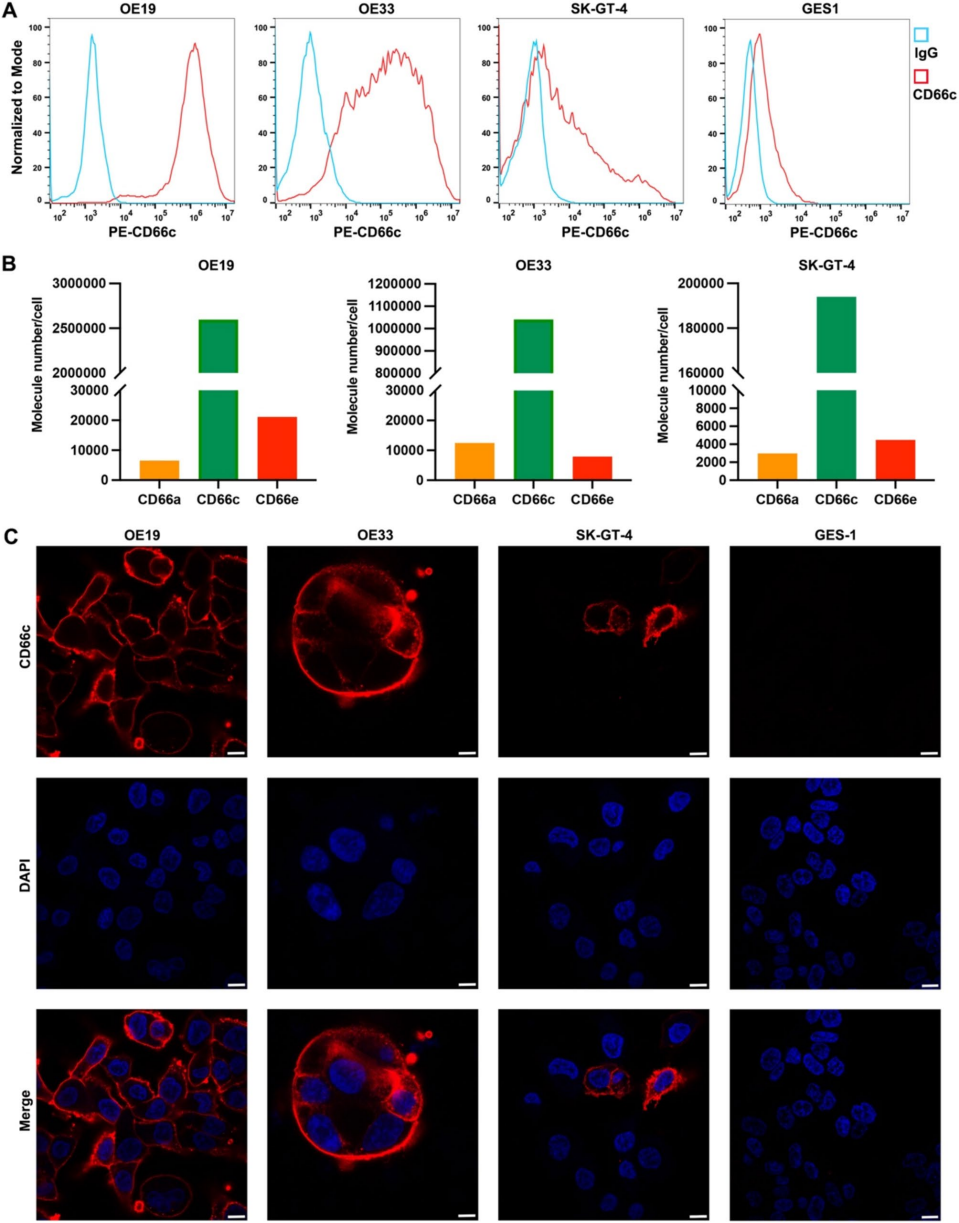
CD66c is a potential ADC target for GEJ cancer. **A** Human GEJ cancer cells and normal GES-1 surface expression of CD66c by flow cytometry (PE-labeled antibody); **B** representative flow cytometry plots showing CD66a, CD66c, and CD66e expression in OE19, OE33, and SK-GT-4 cells; **C** IF staining of CD66c in OE19, OE33, and SK-GT-4 cells

**Fig. 5:**
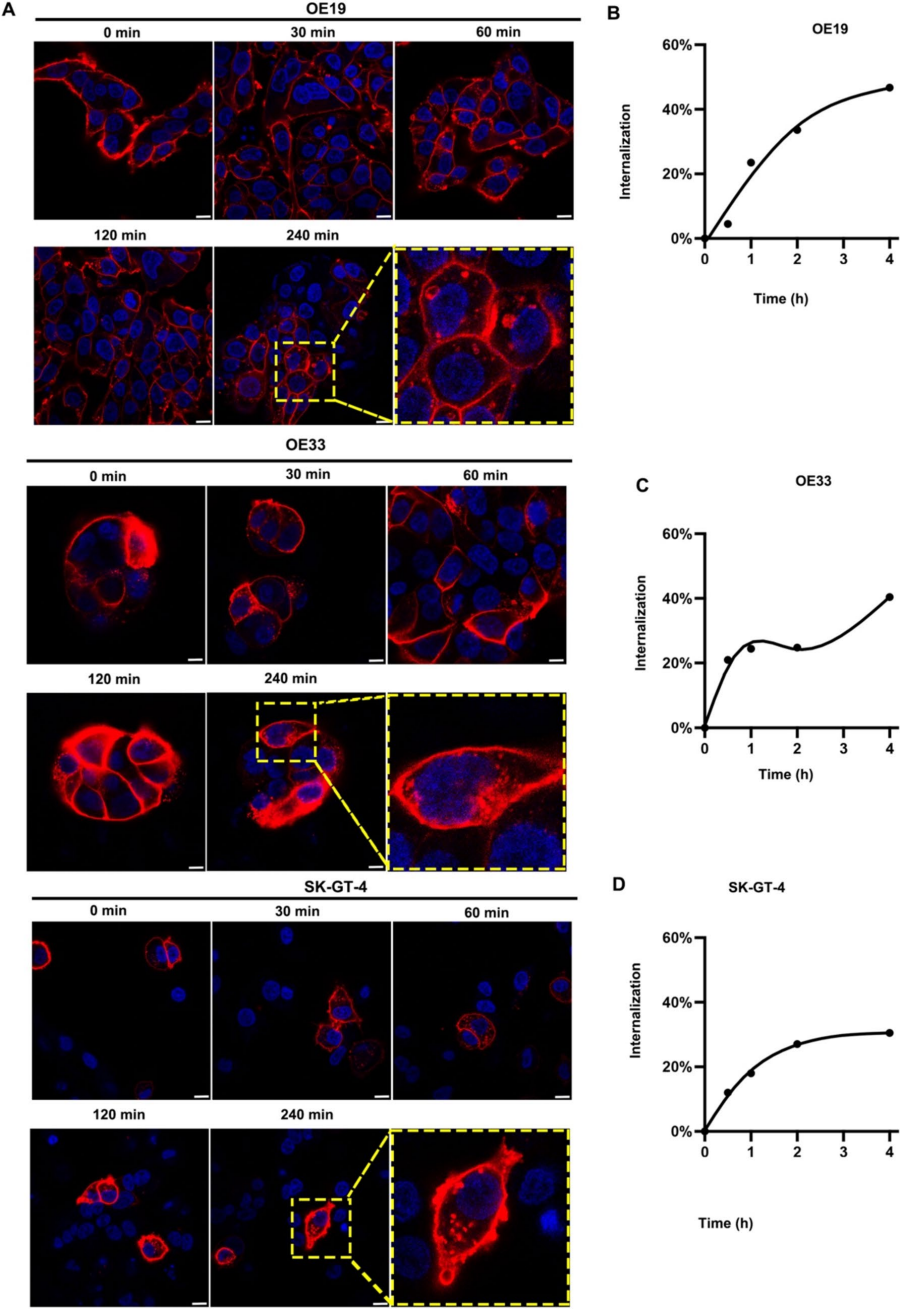
PE-conjugated CD66c antibodies was significantly internalized by GEJ cancer cells. **A** Representative images of IF staining of PE-CD66c antibody internalization in different time point (0 min, 30 min, 60 min, 120 min, and 240 min) in OE19, OE33 and SK-GT-4 cells; **B** internalization curve of PE-CD66c antibody in OE19, OE33, and SK-GT-4 cells quantified by flow cytometry

We investigated the cell membrane expression of CD66c on two human GEJ cancer cell lines (OE19, OE33, and SK-GT-4) and a normal gastric epithelial cell line (GES1) using flow cytometry. As shown in Fig. 4A, OE19 cells exhibited high levels of CD66c expression, while OE33 and SK-GT-4 cells showed moderate expression. In contrast, CD66c expression was undetectable in the normal human epithelial cell line GES1. Additionally, we compared the surface densities of various CEACAM family members including CD66a (CEACAM1), CD66c (CEACAM6), and CD66e (CEACAM5) in the GEJ cell lines. Among these, CD66c displayed the highest expression levels in both OE19 and SK-GT-4 cells (Fig. 4B). Immunofluorescence (IF) staining further confirmed the presence of CD66c on the cytoplasmic membrane of GEJ cells (Fig. 4C). Given the importance of antigen-specific internalization in the efficacy of ADCs [24], we quantitatively assessed the internalization activity of CD66c antibodies in GEJ cancer cells using two independent methods: time-dependent IF staining and flow cytometry. IF staining revealed that PE-conjugated CD66c antibodies initially bound to the cytoplasmic membrane of OE19, OE33, and SK-GT-4 cells and subsequently internalized into endosomes and lysosomes through antigen-mediated endocytosis over time (Fig. 5A). Flow cytometry analysis quantified the internalization kinetics of CD66c antibodies, showing that after 4 h of incubation, the internalization rates plateaued at 46.7% for OE19 cells (Fig. 5B), 40.4% for OE33 cells (Fig. 5C), and 30.5% for SK-GT-4 cells (Fig. 5D). These findings suggest that CD66c antibodies are efficiently internalized by GEJ cancer cells, highlighting CD66c as a promising target for ADC therapy.

### Design, synthesis, and characterization of CD66c-DXd

**Fig. 6:**
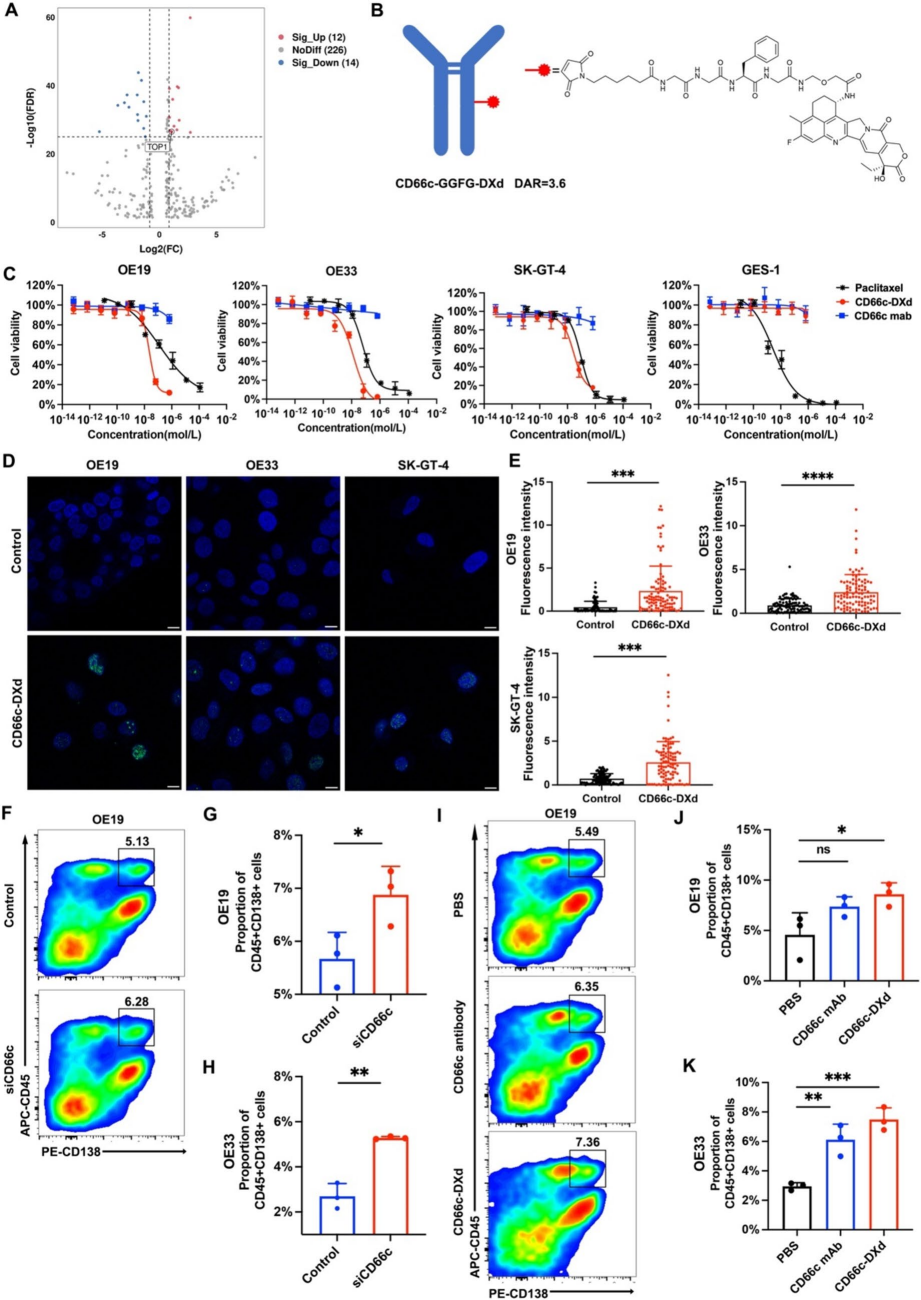
Synthesis of CD66c-DXd and in vitro efficacy of CD66c-DXd. **A** Volcano map of 252 potential candidate drugs in GEJ; **B** schematic illustration and chemical structures of CD66c-DXd linkers and warheads; **C** in vitro cell growth inhibitory activity in OE19, SK-GT-4, and GES-1 cells; **D** representative images of IF staining of pH2AX in CD66c-DXd and control groups. Scale bar 10 μm; **E** fluorescence intensity analysis of pH2AX in CD66c-DXd and control groups in OE19, OE33, and SK-GT-4 cells (*n* = 100 cells per group); **F** representative flow cytometry plots of CD45+CD138+ cells in co-culture of OE19 and PBMCs among control and siCD66c group; quantification of CD45+CD138+ cells in co-culture of OE19 **G** or OE33 **H** and PBMCs among Control and siCD66c group; **I** representative flow cytometry plots of CD45+CD138+ cells in co-culture of OE19 and PBMCs among PBS, CD66c mab, and CD66c-DXd group; quantification of CD45+CD138+ cells in co-culture of OE19 (**J**) or OE33 (**K**) and PBMCs among PBS, CD66c mab, and CD66c-DXd group; * *p* < 0.05; ** *p* < 0.01; *** *p* < 0.001

To identify an efficient payload for advanced GEJ cancer, we conducted an unbiased screening of potential candidate drugs based on the GEJ cancer proteomics. First, the DEPs identified by the proteomics of GEJ cancer were searched in datasets of the Genomics of Drug Sensitivity in Cancer (GDSC), Cancer Therapeutics Response Portal (CTRP), and Broad Institute Drug Repurposing project [35–37]. This integrative approach identified 252 DEPs targeted by FDA-approved drugs or drug candidates currently in clinical trials. Figure 6A presents a volcano plot of these 252 DEPs. The top 10 upregulated DEPs which were potential ADC payloads, based on false discovery rate (FDR) *p* values, were NNMT, CTSB, TYMP, NAMPT, MMP14, GLS, COL1A1, TOP1, CDK1, and PLAUR. Considering the accessibility of ADC payloads, we selected the potent TOP1 inhibitor, DXd, and its corresponding linker (GGFG) for our optimized ADC formulation. We subsequently synthesized CD66c-GGFG-DXd (CD66c-DXd) via cysteine-maleimide conjugation (Fig. 6B). The drug-to-antibody ratio (DAR) of CD66c-DXd was determined to be 3.6 using MALDI-TOF mass spectrometry. We next determined the in vitro toxicity of CD66c-DXd by quantifying their half maximum inhibitory concentrations (IC50s) in GEJ cell lines (Fig. 6C). The IC50s of CD66c-DXd for OE19, OE33, and SK-GT-4 cells were 22.8 nM, 13.3 nM, and 28.4 nM, respectively. Notably, the IC50 of CD66c-DXd was approximately five fold lower than that of paclitaxel, which is currently recommended for treating advanced GEJ cancer according to NCCN guideline. As a CD66c-negtive control, non-neoplastic GES1 cells shows no cytotoxicity when treated with CD66c-DXd due to the lack of CD66c expression. To further validate the cytotoxic efficacy of CD66c-DXd in GEJ cells, DNA damage was assessed using pH2AX staining, a marker for DNA double-strand breaks. Figure 6D and E shows that fluorescence intensity of pH2AX significantly increased after CD66c-DXd treatment compared to the PBS group in OE19 (*p* < 0.001), OE33 (*p* < 0.001), and SK-GT-4 cells (*p* < 0.001). As previously described negative correlation between CD66c and plasma cells (Figs. 2, 3), the OE19 and OE33 cells were co-cultured with PBMCs to investigate the cross talk between CD66c and plasma cells. As is shown in Fig. 6F–H, the proportion of CD45+CD138+ cells was significantly upregulated by CD66c-specific siRNA in OE19 and OE33 cells (OE19: 6.87% vs. 5.67%; *p* = 0.046; OE33: 5.28% vs. 2.69%; *p* = 0.001). To investigate the effect of CD66c antibody and CD66c-DXd on differentiation of B cells into plasma cells, CD66c antibody or CD66c-DXd were added into the co-culture model of OE19 or OE33 cells with PBMCs. Figure 6I–K reveals that the addition of CD66c-DXd significantly upregulated the proportion of CD45+CD138+ cells. These findings demonstrate that CD66c-DXd is a potent and specific therapeutic candidate for GEJ cancer, exhibiting significant cytotoxicity and immunoregulatory effect in GEJ cancer cell lines.

### In vivo therapeutic potential of CD66c-DXd in GEJ cancer treatments

**Fig. 7:**
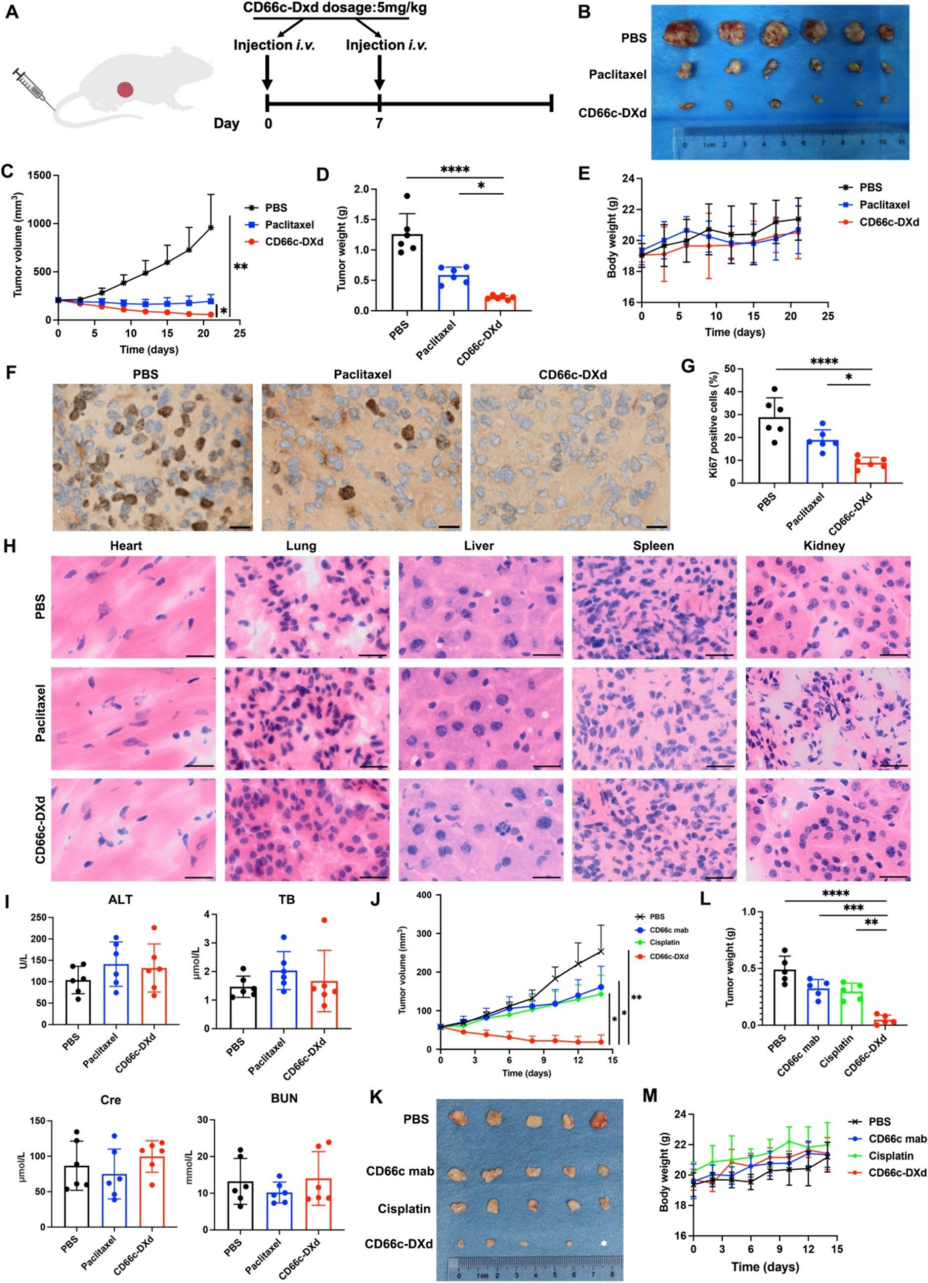
In vivo therapeutic potential of CD66c-DXd in GEJ treatments. **A** Schematic diagram of the GEJ cancer model injected OE19 cells, receiving PBS, paclitaxel, or CD66c-DXd at dosage of 5 mg/kg/week via tail vein injection; **B** image of excised subcutaneous tumors from mice treated with PBS, paclitaxel, and CD66c-DXd (*n* = 6 per group); **C** tumor progression in subcutaneous OE19 tumors by tumor volume measurement by caliper; **D** tumor mass at end point of subcutaneous OE19 tumors were quantified by weight; **E** mouse body weights of subcutaneous OE19 tumors; **F** representative Ki67 IHC staining image of tumor in PBS, paclitaxel, and CD66c-DXd groups. Scale bar 20 μm; **G** quantitation of Ki67-positive cell proportion in PBS, paclitaxel, and CD66c-DXd groups; **H** HE staining of major organs, including heart, lung, liver, spleen, and kidney in PBS, paclitaxel, and CD66c-DXd groups. Scale bar 20 μm; **I** blood chemistry parameters (liver function: ALT and TB; kidney function: Cre and BUN) measured in PBS, paclitaxel, and CD66c-DXd groups; **J** tumor progression in subcutaneous OE33 tumors by tumor volume measurement by caliper; **K** image of excised subcutaneous OE33 tumors from mice treated with PBS, CD66c mab, Cisplatin, and CD66c-DXd (*n* = 5 per group); **L** tumor mass at end point of subcutaneous OE33 tumors were quantified by weight; **M** mouse body weights of subcutaneous OE33 tumors. * *p* < 0.05; ** *p* < 0.01; *** *p* < 0.001

We evaluated the in vivo efficacy of CD66c-DXd using a subcutaneous GEJ xenograft mouse model with OE19 and OE33 cells. Treatment was initiated with intravenous administration of CD66c-DXd at a dosage of 5 mg/kg/week via tail vein injection. Control groups consisted of tumor-bearing mice treated with PBS (sham) or paclitaxel at the same dosage and schedule (Fig. 7A). As demonstrated in Fig. 7B and C, the CD66c-DXd group exhibited significant and sustained tumor regression throughout the experiment. The difference in tumor size between the CD66c-DXd group and the control groups was statistically significant (PBS vs. CD66c-DXd, adjusted *p* = 0.004; Paclitaxel vs. CD66c-DXd, adjusted *p* = 0.011; Fig. 7C). The mean tumor weights were quantified as 1.26 g (PBS), 0.59 g (Paclitaxel), and 0.21 g (CD66c-DXd), respectively (Fig. 7D). Importantly, no obvious bodyweight difference was observed among the three treatment groups (Fig. 7E), suggesting that CD66c-DXd exhibited limited toxicity and was well tolerated in mouse. We performed immunohistochemical (IHC) staining for Ki67, a marker of cell proliferation, in subcutaneous xenografts to evaluate tumor cell proliferation across different treatment groups. The PBS-treated group exhibited numerous Ki67-positive cells, indicating high levels of cell proliferation. In contrast, the CD66c-DXd-treated group showed significantly fewer Ki67-positive cells, suggesting a potent inhibitory effect on tumor growth (Fig. 7F). Statistical analysis confirmed the anti-proliferative effects of CD66c-DXd (PBS vs. CD66c-DXd, adjusted *p* < 0.001; Paclitaxel vs. CD66c-DXd, adjusted *p* = 0.025; Fig. 7G). Treatment-related toxicities to major organs (heart, lung, liver, spleen, and kidney) were evaluated by histology analysis with HE staining. The HE staining revealed no apparent drug treatment-related necrosis or degenerative changes in these organs across all treatment groups (Fig. 7H). Additionally, serum biochemical parameters were measured to evaluate organ function and potential toxicity of the ADC. Liver function markers (ALT and TB) and kidney function markers (Cre and BUN) showed no significant differences among the three groups, indicating no evident off-target effects in vivo (Fig. 7I). The potent in vivo efficacy of CD66c-DXd in GEJ cancer was confirmed with OE33 xenograft mouse model. The treatment schedule was same with Fig. 7A. As is shown in Fig. 7J and K, the CD66c-DXd group exhibited significant tumor regression in comparison with PBS, CD66c mab and cisplatin group. The comparison of tumor weight achieved the similar results (Fig. 7L). The bodyweight among the different groups was similar (Fig. 7M). These findings underscore the efficacy and safety of CD66c-DXd in targeting GEJ tumors while minimizing systemic toxicity.

**Fig. 8:**
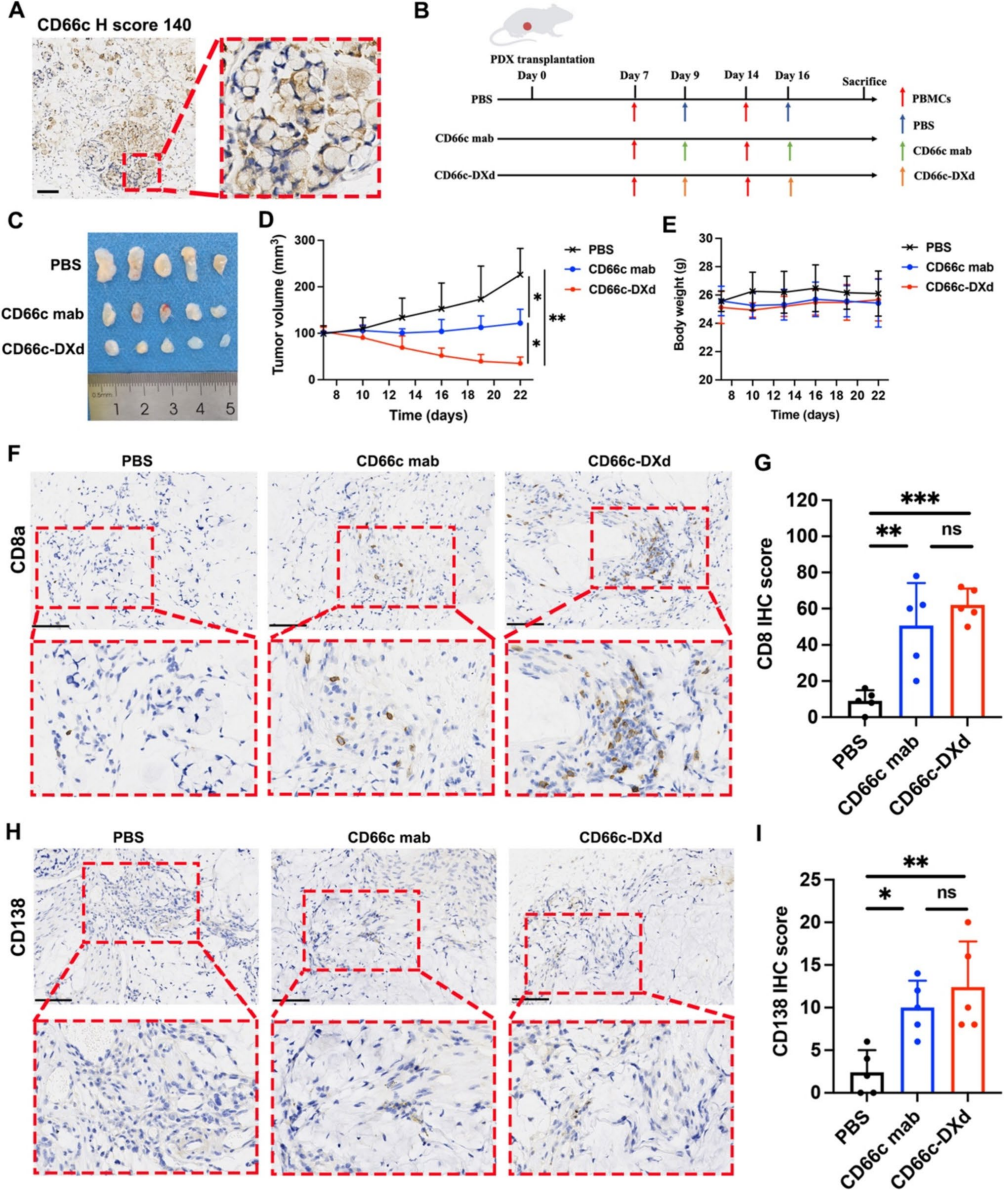
CD66c-DXd showed potent anti-tumor efficacy and effective immunotherapeutic capacity in PDX model of GEJ cancer. **A** Representative images of IHC staining for CD66c in GEJ caner PDX model. Scale bar 100 μm; **B** schematic design of PDX tumor in NCG mice; **C** image of excised subcutaneous PDX tumors from mice treated with PBS (sham), CD66c mab or CD66c-DXd group (*n* = 5 per group); **D** tumor progression curve of PDX in NCG mice by tumor volume measurement by caliper; **E** quantified NCG mice weight in different groups; **F** representative images of IHC staining for CD8a in PDX tissue samples among different groups, Scale bar 100 μm; **G** IHC H-score of CD8a among different groups; **H** representative images of IHC staining for CD138 in PDX tissue samples among different groups, scale bar 100 μm; **I** IHC H-score of CD138 among different groups; Bonferroni-adjusted *p* value <0.05 was considered statistically significant

Furthermore, we also built patient-derived xenografts (PDX) of GEJ cancer in NCG mice to evaluate the in vivo efficacy of CD66c-DXd. The CD66c H-score of PDX was 140 (Fig. 8A). Fig. 8B illustrates the drug dosage and schedule. The PBMCs were co-administrated to assess the immunoregulatory effect of CD66c-DXd in vivo. As is shown in Fig. 8C and D, the CD66c-DXd group significantly decreased the tumor volume. The bodyweight among the different groups was similar (Fig. 8E). We performed the IHC of CD8a and CD138. As is shown in Fig. 8F and G, the CD66c-DXd group significantly increased CD8+ T-cell infiltration in comparison with PBS group (Berforroni-adjusted *p* = 0.001). The CD138+ plasma cells were also upregulated in CD66c-DXd group in comparison with PBS group (Berforroni-adjusted *p* = 0.004). Taken together, we confirmed that CD66c-DXd possessed potent cytotoxic activity and immunoregulatory effect in GEJ cancer.

## Discussion

In this study, we conducted a comprehensive multi-omics analysis of GEJ cancer to identify the membrane target CD66c and synthesized the corresponding ADC drug, CD66c-DXd. Our findings demonstrate that CD66c-DXd exhibits significant anti-tumor efficacy with limited toxicity in multiple GEJ tumor models. This study provides a paradigm for the identification of specific membrane targets and the synthesis of ADCs with tailored payloads for particular cancers, advancing the development of targeted cancer therapies.

GEJ cancer patients typically face a poor prognosis, exacerbated by a lack of GEJ specific clinical trial data evaluating therapeutic agents within the broader category of gastric cancers. ADCs represent a promising therapeutic strategy in cancer treatment. Utilizing the specificity of antibodies to deliver potent cytotoxic drugs directly to tumor cells, potentially enhancing efficacy and reducing systemic toxicity. Continued research and development in the field of ADCs hold great promise for the future of precision cancer medicine. A major hurdle in the pursuit of GEJ-targeted ADC drugs is the identification of suitable membrane targets that can effectively distinguish between malignant GEJ cells and normal tissues. Our results demonstrated that CD66c is a novel ADC target for GEJ cancer due to its high tumoral overexpression and effective cell internalization. CD66c, also known as CEACAM6, is a multifunctional glycoprotein located at the 19q13.2 locus of the human genome, mediating interactions with integrin receptors. Previous studies have shown that CD66c levels are associated with tumor progression and metastasis in various cancer types [38, 39]. Overexpression of CD66c has been linked to key cancer hallmarks, including abnormal cell differentiation, resistance to apoptosis, cell proliferation, and resistance to therapeutic agents [38]. Pandey et al. further elucidated the potential impact of CD66c knockout in pancreatic cancer cell line (HPAF-II) and found that knockout of CD66c could profoundly impact extracellular matrix (ECM) functions including cell adhesion, immune environment, autophagy, DNA repair, chromatin modifications, and signal transduction [40].

Notably, previous studies demonstrated that CD66c significantly influenced tumor immune microenvironment (TIME) through its interactions with integrins and immune cells [41]. Specifically, antibody-mediated cross-linking of CD66c activates neutrophils, promoting their adhesion to the ECM and enhancing T-cell-mediated elimination of cancer cells [27, 41]. Furthermore, Pinkert et al. showed that CD66c antibody increased cytokine secretion by T cells and enhanced T-cell-mediated killing of cancer cells [27]. The anti-tumor activity of CD66c antibodies was further amplified when combined with anti-PD-1 or anti-TIM3 checkpoint blockade therapies [27]. In our study, the CIBERSORT deconvolution analysis revealed that high CD66c expression was associated with a significantly lower proportion of plasma cells. This finding is noteworthy, because previous research by Patil et al. indicated that the intratumoral plasma cells could accurately predict outcomes to PD-L1 blockade in non-small cell lung cancer, showing a strong correlation between plasma cell abundance and overall survival specific to PD-L1 blockade [42]. Additionally, patients exhibiting high expression of B-cell gene signatures have shown significantly favorable prognosis across multiple solid tumors in The Cancer Genome Atlas (TCGA) cohort, irrespective of treatment modality [43, 44]. This is likely due to the plasma cells’ ability to produce tumor-antigen-specific antibodies, thereby leading to complement activation and antibody-dependent cellular cytotoxicity [45]. Moreover, CEACAM1, a known ligand of CD66c, has been reported as a potent regulator of B-cell receptor complex-induced activation [46]. Collectively, these findings suggest that blockade of CEACAM6 may potentially remodel the TIME, thereby unleashing potent anti-tumor immunity. Thus, targeting CD66c axis may offer a promising strategy to enhance the efficacy of immunotherapies.

In this study, we demonstrated that CD66c is a promising ADC target for GEJ cancer. Notably, the expression level and tumor specificity of CD66c in GEJ cancer are significantly higher than other established ADC targets, including HER2, CLDN18.2, and TROP2. Further, specificity of efficacy was determined in a therapy experiment in the CD66c-negative cell line (GES1). Recent studies have reported CD66c as an efficient target in CAR-T cell therapy for pancreatic cancer, highlighting its specific targeting capabilities with limited distribution in normal tissues [47]. Furthermore, we describe a comprehensive strategy for ADC target identification and efficient payload selection by leveraging multi-omics data. Large-scale multi-omics data, which account for human complexity and encompass multicomponent biomarker panels including genetic, personal, and environmental factors, provide crucial insights to guide the rational selection of optimal ADC targets and payloads in drug discovery [48]. The encouraging preclinical data obtained with the CD66c-DXd in GEJ cancer pave the way for the exploration of its potential use in the clinical setting.

Despite the encouraging findings in this study, there are several limitations that warrant further investigation. The lack of expression of a murine homologue of CD66c precludes preclinical exploration of the immunomodulatory function of CD66c-DXd in vivo. To address this constraint, the human CD66c-transgenic mouse was needed to assess the immunomodulatory function of CD66c-DXd. This limitation also impedes a fair assessment of the potential toxicities of CD66c-DXd due to its specific action on endogenous CD66c. The potential anti-tumor immunity, pharmacokinetics, and systemic toxicity of CD66c-DXd are also needed to explore in large animals and non-human primates, that more accurately mirror human responses. The patient-derived xenograft (PDX) model should also be applied to further research to heterogeneous GEJ cancers.

## Conclusion

In this study, we developed a robust and generalizable screening approach to identify ADC membrane targets for tumor-targeted therapy. Through a multi-omics integrative analysis encompassing transcriptomics, proteomics, and phosphoproteomics, we determined the potential of CD66c as a promising ADC target for advanced GEJ cancer. This comprehensive approach also included evaluating receptor-mediated internalization and ADC efficacy, culminating in the first demonstration of the anti-tumor efficacy of CD66c-DXd in preclinical models of GEJ cancer. The encouraging experimental evidence supports the potential of CD66c-DXd as a promising ADC candidate for GEJ cancer, warranting further clinical investigations.

## Data Availability

All data relevant to the study are included in the article.
